# Radiation quality-dependence of bystander effect in unirradiated fibroblasts is associated with TGF-β1-Smad2 pathway and miR-21 in irradiated keratinocytes

**DOI:** 10.1038/srep11373

**Published:** 2015-06-16

**Authors:** Xiaoming Yin, Wenqian Tian, Longxiao Wang, Jingdong Wang, Shuyu Zhang, Jianping Cao, Hongying Yang

**Affiliations:** 1 School of Radiation Medicine and Protection, Medical College of Soochow University/Collaborative Innovation Center of Radiation Medicine of Jiangsu Higher Education Institutions, 199 Renai Road, Suzhou Industrial Park, Suzhou, Jiangsu Province, P. R. China 215123

## Abstract

Traditional radiation biology states that radiation causes damage only in cells traversed by ionizing radiation. But radiation-induced bystander effect (RIBE), which refers to the biological responses in unirradiated cells when the neighboring cells are exposed to radiation, challenged this old dogma and has become a new paradigm of this field. By nature, RIBEs are the consequences of intercellular communication between irradiated and unirradiated cells. However, there are still some important questions remain unanswered such as whether RIBE is dependent on radiation quality, what are the determining factors if so, etc. Using a transwell co-culture system, we found that HaCaT keratinocytes irradiated with α-particles but not X-rays could induce bystander micronucleus formation in unirradiated WS1 fibroblasts after co-culture. More importantly, the activation of TGF-β1-Smad2 pathway and the consistent decrease of miR-21 level in α-irradiated HaCaT cells were essential to the micronucleus induction in bystander WS1 cells. On the other hand, X-irradiation did not induce bystander effect in unirradiated WS1 cells, accompanied by lack of Smad2 activation and consistent decrease of miR-21 in X-irradiated HaCaT cells. Taken together, these results suggest that the radiation quality-dependence of bystander effect may be associated with the TGF-β1-Smad2 pathway and miR-21 in irradiated cells.

Non-targeted effects, which include low dose hypersensitivity, genomic instability, radiation-induced adaptive response and radiation-induced bystander effect (RIBE), have now become new dogmas in radiation biology^1^. Among them, RIBE refers to the biological alterations such as DNA damage, cell killing, gene expression, mutation etc. in unirradiated cells when the neighboring cells are traversed by ionizing radiation. So far, although RIBE has been demonstrated in various types of cells exposed to different types of radiation^2^,^3^,^4^,^5^,^6^,^7^,^8^,^9^,^10^, it is still controversial whether RIBE is a universal phenomenon^11^,^12^. In addition to the proposed factors such as the epigenetic status of a specific cell line, the precise culture conditions, medium supplements and appropriate endpoint detected at appropriate time^11^,^12^,^13^, radiation quality may be another factor that is critical to the occurrence of RIBE. Recently several studies have shown that short-term and long-term RIBEs are dependent on radiation quality^14^,^15^,^16^,^17^. However, no detailed explanation has been provided.

The molecular mechanisms underlying RIBE have been one of the hot topics in radiation biology since 1992, when Nagasawa and Little directly demonstrated the occurrence of RIBE^5^. RIBEs are the consequences of intercellular communication by nature, which can be mediated through intercellular gap junctions^18^,^19^, reactive oxygen species (ROS)^8^,^20^ and soluble signaling molecules such as cytokines^21^,^22^. For example, tumor growth factor β1 (TGF-β1) has been found to be one of the RIBE mediators^13^,^23^,^24^,^25^,^26^. In addition to the bystander signaling molecules, both the ionizing radiation-induced signaling pathways in irradiated cells that result in the release of signaling molecules and the pathways in unirradiated cells that are activated by the signaling molecules are important to the initiation of RIBEs. BRCA1, FANCD2 and Chk1 have been found to be the potential targets for the modulation of radiation response in bystander cells^27^. iNOS-NO signaling in irradiated cells and p38 pathway in unirradiated cells have been demonstrated to play important roles in RIBEs^28^. Our previous study has shown that the TGF-β1 signaling pathways in both irradiated and bystander cells are critical to the induction of bystander effects^13^. In the canonical TGF-β1 signaling pathway, Type II TGF-β receptor (TGFBR2) binds to TGF-β1 ligand, then forms a heterodimer with Type I TGF-β receptor (TGFBR1) and activates/phosphorylates Smad2/Smad3, eventually inducing Smad4-dependent transaction. And Smad7 negatively regulates the activation of Smad2/Smad3^29^. Ionizing radiation activates TGF-β-Smad pathways^30^. Both Smad2 and Smad7 have been found to play an important role in radiation-induced double strand break (DSB) signaling^31^. However, it remains undefined whether the activation of TGF-β1/Smad signaling pathways in irradiated cells leading to RIBEs depends on radiation quality.

The roles of microRNA (miRNA) in RIBEs have been actively investigated recently. Although the study from Dickey *et al.* suggests that instead of a primary signaling factor, the changes in the expression of miRNAs are more likely a manifestation of RIBE^32^, we and others have demonstrated an important mediating role of miRNAs such as miR-21 and miR-663^13^,^33^,^34^. These results support the hypothesis that bystander effect is epigenetically mediated^35^,^36^,^37^. However, it is still not clear how miRNAs mediate RIBEs. Due to the different roles of irradiated and unirradiated cells in RIBEs, it is possible that miRNAs in these two cell populations mediate RIBEs through different mechanisms. For irradiated cells, the miRNA profiles of cells undergo different changes upon different types of radiation^38^. Nevertheless, it remains unclear whether the dependence of RIBEs on radiation quality is related to the different changes of miRNAs.

More interestingly, some miRNAs such as miR-21 execute their functions through modulation of TGF-β1 signaling pathways. For example, miR-21 plays a critical role in the induction of carcinoma-associated fibroblasts by regulating TGF-β1 signaling through its direct target, Smad7^29^. miR-21 participates in cardiac fibrosis via its reciprocal interaction with its another target, TGF-β receptor III (TGFBRIII)^39^. And miR-21 regulates the differentiation of human adipose tissue-derived mesenchymal stem cells by targeting TGFBR2 and altering the phosphorylation of Smad3^40^. On the other side, activated Smad2 and 3 are critically involved in the post-transcriptional processing of miR-21 41,42. These results suggest that miR-21 and TGF-β1-Smad pathways can regulate each other. More recently, miR-663 has been found to inhibit RIBEs by targeting TGF-β1 in a feedback mode^33^. Thus it would be interesting to investigate the relationship between miR-21 and TGF-β1 pathways in irradiated cells sending bystander signals.

In the present study, by using a transwell insert co-culture system, we investigated medium-mediated bystander micronucleus (MN) formation in unirradiated skin fibroblasts after co-cultured with skin keratinocytes exposed to α-particles or X-rays. More importantly, we explored the roles of TGF-β1-Smad2 signaling pathway and miR-21 of irradiated keratinocytes in the micronucleus formation of bystander fibroblasts and the relationship between miR-21 and TGF-β1-Smad2 signaling, and aimed to elucidate the reason why RIBE is associated with radiation type.

## Results

### HaCaT cells irradiated with α-particles but not X-rays could induce MN formation in unirradiated bystander WS1 cells

**Figure 1:**
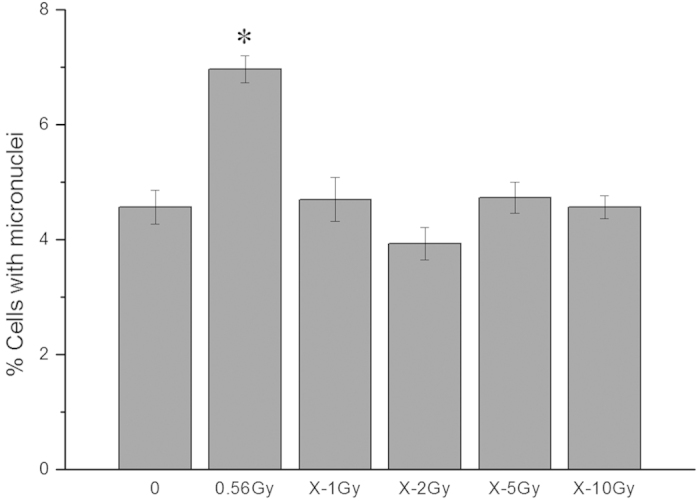
Unirradiated WS1 cells displayed bystander micronucleus formation after co-cultured with α-irradiated HaCaT cells but not X-irradiated HaCaT cells for 24 h. * P < 0.05 compared with the WS1 cells co-cultured with sham irradiated HaCaT cells.

**Figure 2:**
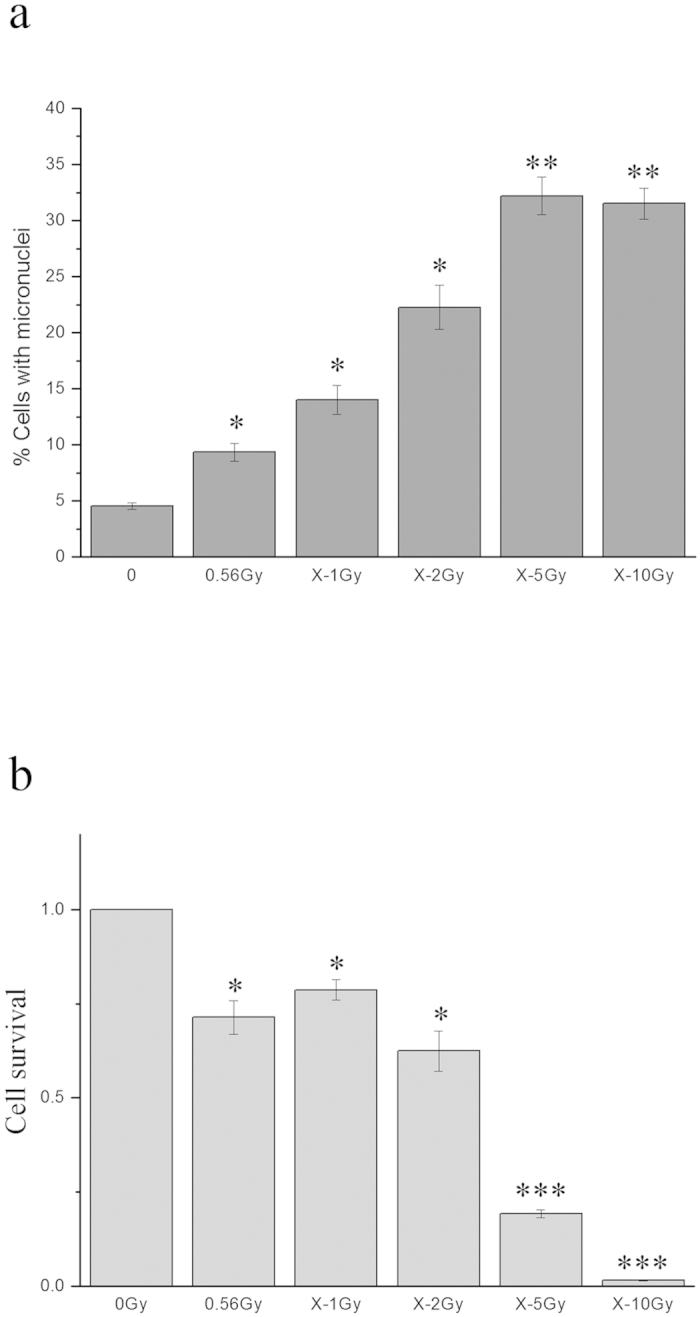
X-irradiation caused more serious damage and cell killing in directly irradiated HaCaT cells than 56 cGy of α-particles did. Panel **a**, the micronucleus formation in HaCaT cells 48 h after irradiation. Panel **b**, the cell survival of irradiated HaCaT cells. * P < 0.05, ** P < 0.01, *** P < 0.001 compared with the sham irradiated control.

MN formation in unirradiated WS1 cells was chosen as the endpoint for radiation-induced bystander response. We found that after co-cultured with HaCaT cells irradiated with 56 cGy of α-particles for 24 h, there was a 1.53-fold (1.53 ± 0.08) increase (*P* = 0.011) in the frequency of MN formation in unirradiated bystander WS1 cells compared with the WS1 cells co-cultured with sham irradiated HaCaT cells (Fig. 1), indicating the occurrence of medium-mediated bystander signaling between α-irradiated HaCaT cells and unirradiated WS1 cells, eliciting damage in bystander WS1 cells. Surprisingly, when HaCaT cells were irradiated with 1 Gy of X-rays, no induction of MN in bystander WS1 was observed (*P* = 0.72) (Fig. 1). To determine whether lack of MN formation in WS1 cells after co-cultured with X-irradiated HaCaT cells was due to the dose used, which was 1 Gy, we also irradiated HaCaT cells with 2, 5 or 10 Gy of X-rays, and still did not observe an increase in the MN frequency in unirradiated WS1 cells after co-culture (Fig. 1), although X-irradiation caused significantly greater MN formation and cell killing in irradiated HaCaT cells at used doses than 56 cGy of α-particles did (Fig. 2a,b). These results suggest that radiation-induced bystander response between HaCaT and WS1 cells may be dependent on radiation quality.

### TGF-β1-Smad2 signaling in irradiated HaCaT cells played an important role in the induction of bystander MN formation in unirradiated WS1 cells

**Figure 3:**
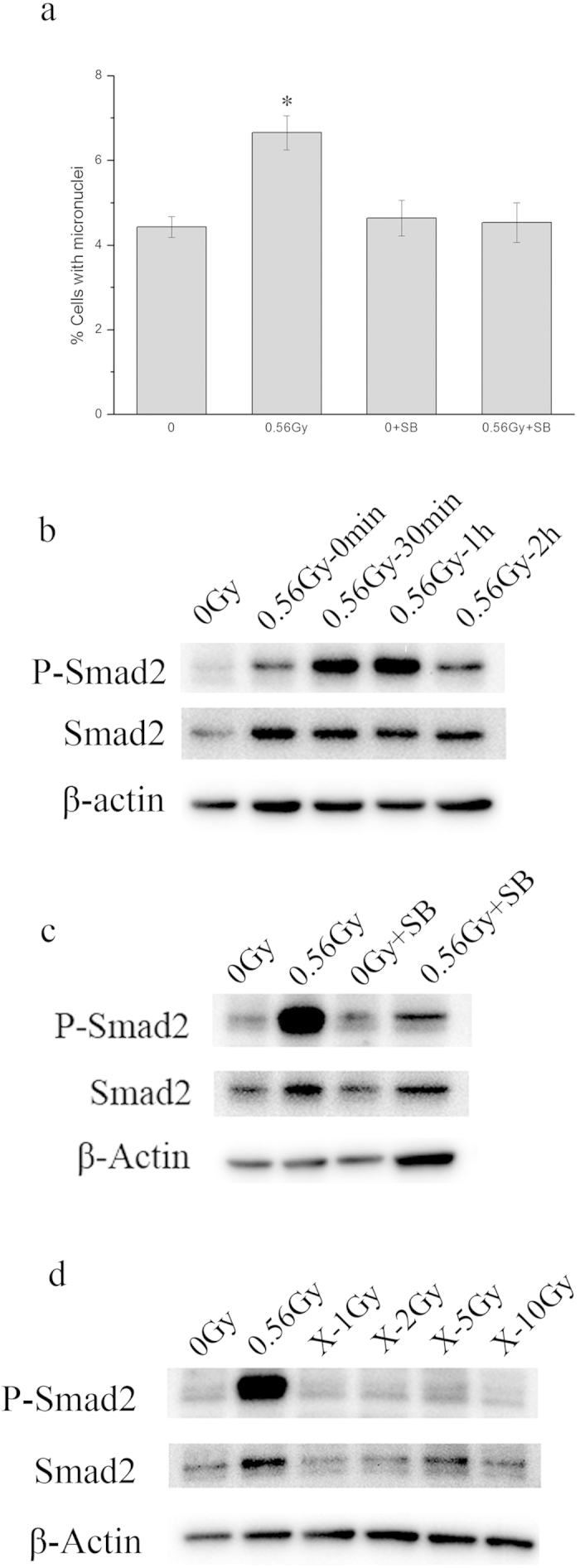
TGF-β1-Smad2 pathway was involved in bystander micronucleus formation in unirradiated WS1 cells co-cultured with α-irradiated HaCaT cells. Panel **a**, the effect of SB431542 on the bystander micronucleus formation in unirradiated WS1 cells. * P <0.05 compared with the WS1 cells co-cultured with sham irradiated HaCaT cells. Panel **b**, the phosphorylation of Smad2 in HaCaT cells at various times after α- irradiation. Panel **c**, the effect of SB431542 on the phosphorylation of Smad2 in HaCaT cells 1 h after α-irradiation. Panel **d**, In contrast with α-irradiation, X-rays did not cause phosphorylation of Smad2 in HaCaT cells 1 h after radiation up to 10 Gy.

Our previous study has demonstrated that the activation of TGF-β1 pathway in irradiated H1299 human lung cancer cells is involved in the medium-mediated bystander signaling^13^, therefore, in the present study we tested whether TGF-β1 signaling was critical to the induction of bystander response in skin cell system, too. HaCaT cells were pretreated with SB431542, a potent and selective inhibitor of TGF-β1 receptor kinases, prior to α-irradiation, then co-cultured with unirradiated WS1 cells after irradiation. And we observed no MN formation in bystander WS1 cells (*P* = 0.74) (Fig. 3a). This suggests that TGF-β1 signaling was activated in α-irradiated HaCaT cells and its inhibition prevented the induction of bystander effect in WS1 cells.

It is known that activated TGF-β receptor activates Smad2. To explore further the involvement of TGF-β1 signaling pathway of irradiated HaCaT cells in bystander response, we detected the activation/phosphorylation of Smad2 in irradiated HaCaT cells. Not unexpectedly, Smad2 in HaCaT cells was phosphorylated immediately after α-irradiation, and the elevated phosphorylation level last for at least 2 hours (Fig. 3b). In addition, when HaCaT cells were treated with SB431542 prior to α-irradiation, α- particle-induced phosphorylation of Smad2 was significantly inhibited (Fig. 3c). Coinciding with the lack of MN formation in bystander WS1 cells after co-cultured with X-irradiated HaCaT cells, Smad2 was not activated after HaCaT cells were irradiated with X-rays (Fig. 3d). These results may suggest that activation of TGF-β1-Smad2 pathway was essential to the induction of bystander response.

### miR-21 in irradiated HaCaT cells was an important mediator of bystander effect

**Figure 4:**
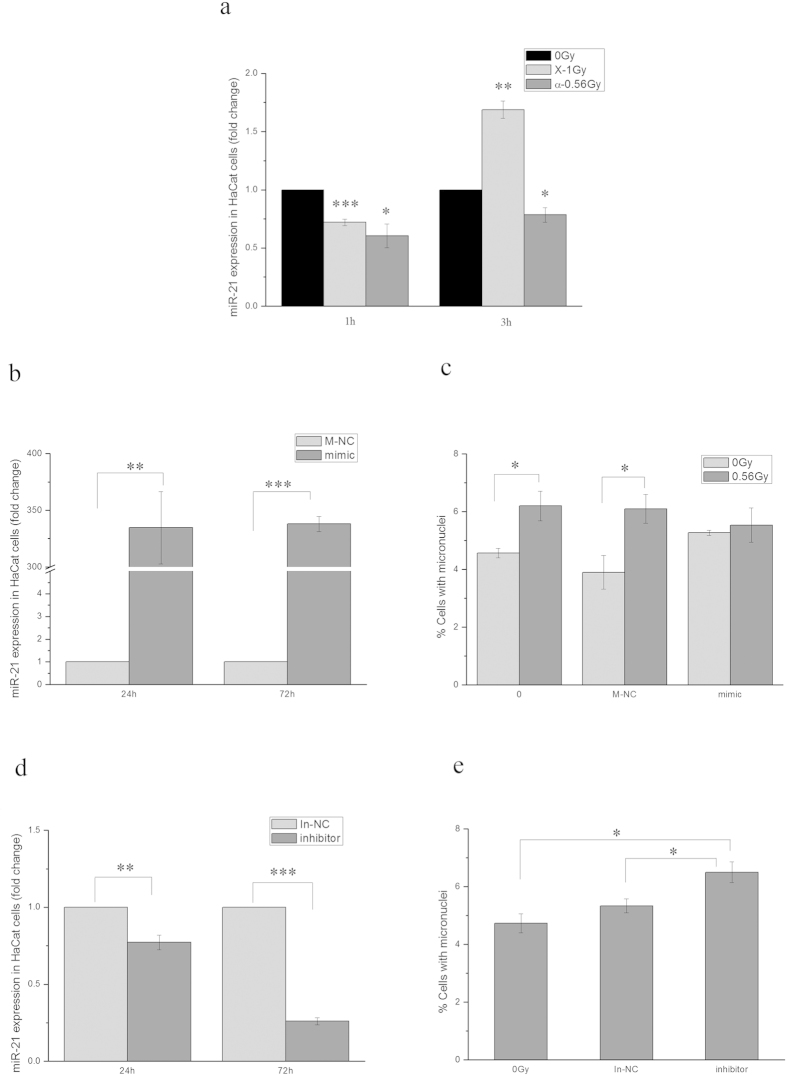
miR-21 played an important role in bystander micronucleus formation in WS1 cells co-cultured with α-irradiated HaCaT cells. Panel **a**, the alterations of miR-21 of HaCaT cells after α- and X-irradiation. Panel **b,** overexpression of miR-21 in HaCaT cells 24 and 72 h after miR-21 mimic transfection. Panel **c**, the effect of overexpression of miR-21 in HaCaT cells by miR-21 mimic transfection on the bystander micronucleus formation in unirradiated WS1 cells. Panel **d**, downregulation of miR-21 in HaCaT cells 24 and 72 h after miR-21 inhibitor transfection. Panel **e**, downregulation of miR-21 in HaCaT cells alone induced bystander-like effect in WS1 cells after co-culture. * P < 0.05, **P < 0.01 and *** P < 0.001 compared with the relative control.

We have shown an important mediating role of miR-21 of unirradiated cells in bystander response in the previous study^13^. To investigate whether miR-21 of irradiated signaling cells was involved in the induction of bystander effect, we first measured the changes of miR-21 expression level in HaCaT cells after irradiation. When HaCaT cells were irradiated with 1 Gy of X-rays, the expression level of miR-21 decreased about 1.39 ± 0.03 folds (*P* = 0.00028) 1 h after irradiation, but increased 1.69 ± 0.07 folds (*P* = 0.0027) 3 h after irradiation. In contrast, when HaCaT cells were irradiated with 56 cGy of α-particles, miR-21 expression decreased about 1.66 ± 0.10 (*P* = 0.037) and 1.27 ± 0.06 folds (*P* = 0.042) at 1 and 3 h after irradiation, respectively (Fig. 4a). The fact that X-rays and α-particles induced different alterations of miR-21 expression suggests that different types of radiation induce different gene expression and signaling.

To determine whether the consistent decrease of miR-21 expression in HaCaT cells after α-irradiation was critical to the induction of bystander response in unirradiated WS1 cells, we overexpressed miR-21 in HaCaT cells by transfecting the cells with miR-21 mimic, then irradiated them with α-particles. Figure 4b shows that the transfection of miR-21 mimic led to a over 300-fold increase (334.63 ± 31.94) (*P* = 0.0090) in miR-21 expression at 24 h after transfection and last for another 48 h. Furthermore, no increase in MN frequency (*P* = 0.65) in bystander WS1 cells was observed after co-cultured with the miR-21-overexpressed HaCaT cells irradiated with α-particles, which was in contrast with the results with the parental HaCaT cells and the HaCaT cells transfected with negative control for miR-21 mimic (*P* = 0.028) (Fig. 4c). On the other hand, we downregulated miR-21 expression in HaCaT cells by transfecting cells with miR-21 inhibitor, then co-cultured the transfected cells with WS1 cells. We found that the transfection of miR-21 inhibitor resulted in 1.30-fold (1.30 ± 0.05) (*P* = 0.0045) and 3.85-fold (3.85 ± 0.02) (*P* = 0.00097) decrease in miR-21 expression at 24 and 72 h after transfection, respectively (Fig. 4d). And reducing the miR-21 expression in HaCaT cells alone induced a 1.38-fold (1.38 ± 0.08) (*P* = 0.038) and 1.22-fold (1.22 ± 0.03) (*P* = 0.024) increase in MN formation in co-cultured WS1 cells compared with the parental HaCaT cells and the HaCaT cells transfected with the negative control, respectively (Fig. 4e). These results indicate an important mediating role of miR-21 of irradiated HaCaT cells in the induction of bystander response in WS1 cells.

### miR-21 and TGF-β1-Smad2 pathway regulated each other to mediate bystander response

**Figure 5:**
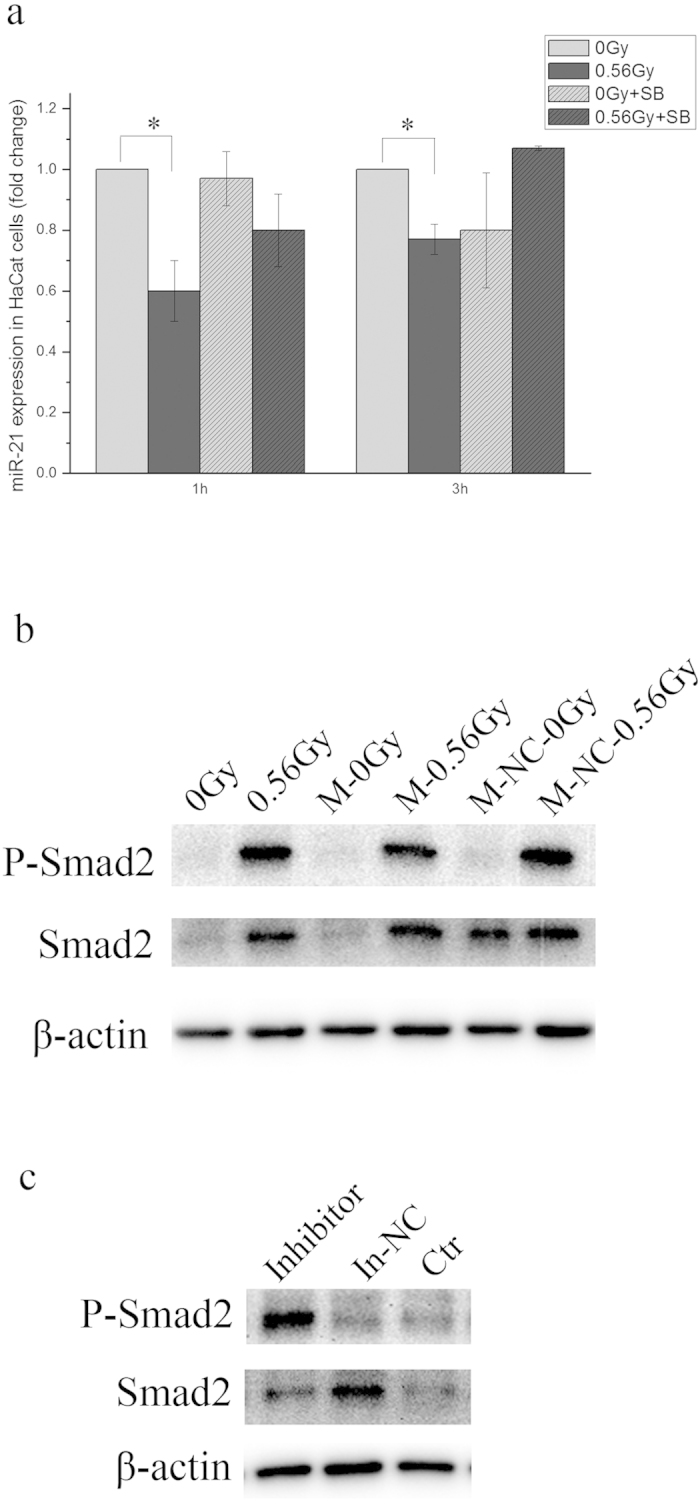
TGF-β1-Smad2 pathway and miR-21 regulated each other. Panel **a**, the effect of SB431542 on the miR-21 expression level in HaCaT cells irradiated with α-particles. * P < 0.05 compared with the relative control. Panel **b**, the effect of overexpression of miR-21 on the phosphorylation of Smad2 in HaCaT cells upon α-irradiation. Panel **c**, downregualtion of miR-21 in HaCaT cells alone induced phosphorylation of Smad2.

Using a transwell insert co-culture system, we have demonstrated the involvement of TGF-β1-Smad2 pathway and miR-21 of α-irradiated HaCaT cells in the bystander MN formation in unirradiated WS1 cells, we then determined the relationship between miR-21 and TGF-β1-Smad2 pathway. On one hand, as shown in Fig. 5a, the pretreatment of HaCaT cells with SB431542 prior to α-irradiation rescued the decrease in miR-21 expression from 1.66 ± 0.10 folds to 1.21 ± 0.12 folds (*P* = 0.173) 1 h after irradiation, and slightly increased the miR-21 expression 3 h after irradiation (*P* = 0.145). Since SB431542 pretreatment significantly decreased the phosphorylation of Smad2 (Fig. 3c), these results suggest that inhibition of TGF-β1-Smad2 signaling affected the alterations of miR-21 expression of HaCaT cells upon α-irradiation. On the other hand, when the miR-21-overexpressed HaCaT cells were irradiated with α-particles, compared with the parental cells and the cells transfected with the negative control of miR-21 mimic, the activation of Smad2 was inhibited (Fig. 5b). Additionally, the phosphorylation of Smad2 was elevated in the HaCaT cells transfected with miR-21 inhibitor compared with in the cells transfected with the negative control for miR-21 inhibitor (Fig. 5c). These results indicate that down regulation of miR-21 alone could induce phosphorylation of Smad2, and overexpression of miR-21 could inhibit α-irradiation-induced activation of Smad2 in HaCaT cells. All of these results suggest that miR-21 and the TGF-β1-Smad2 pathway involved in bystander signaling were not independent, they regulated each other.

## Discussion

Since the demonstration and confirmation of RIBEs, the factors involved in the induction of RIBEs have been intensively explored, especially when there is still some debate regarding the universality of this phenomenon. Radiation quality is among the factors that could affect the occurrence of RIBEs in spite of the contradictory reports on the LET dependence of RIBEs^14^,^15^,^16^,^17^,^43^. In the present study, we found that HaCaT cells irradiated with α-particles but not X-rays could elicit bystander MN formation in unirradiated WS1 cells through medium-mediated mechanisms (Fig. 1), suggesting the radiation quality dependence of bystander MN formation in this system. Although more radiation types are needed to verify this conclusion in the near future, our results are similar to the previous reports^14^,^15^,^16^,^17^ that bystander effects were LET-dependent. Moreover, our results agree with those from the studies of Buonanno *et al.*^14^,^15^ showing an increase in neoplastic transformation frequency, reduced cloning efficiency and increased levels of chromosomal damage, protein oxidation and lipid peroxidation in the progeny of bystander cells co-cultured with cells irradiated with high LET but not low LET irradiation.

Taking a step further, we investigated why bystander MN formation in unirradiated WS1 cells could be induced by α-irradiated but not X-irradiated HaCaT cells. Previous studies of RIBE mainly focused on the signaling molecules released by irradiated cells. But one has to keep it in mind that the bystander signaling molecules are highly dependent on the specific signaling pathways in irradiated cells activated by ionizing radiation. Our previous study^13^ has demonstrated that the TGF-β1 pathway of irradiated cells is critical to the induction of RIBEs. In the present study, we confirmed that conclusion in a different system, suggesting the prevalent role of TGF-β1 pathway of irradiated cells in RIBEs.

We found that Smad2 phosphorylation in irradiated HaCaT cells was involved in initiation of bystander effect. On one hand, Smad2 was rapidly phosphorylated in α-irradiated HaCaT cells (Fig. 3b); On the other hand, when the TGF-β1 pathway of irradiated cells was inhibited with the TGF-βR1 inhibitor, SB431542, the activation of Smad2 was significantly inhibited (Fig. 3c), so as the bystander effect (Fig. 3a). Cytokine signaling pathways such as TGF-β-Smad pathways are activated by ionizing radiation^31^,^44^,^45^,^46^. While phosphorylation of Smad2 was observed in cultured cells or animals irradiated with high doses of X-rays (8 Gy for *in vitro* or above 15 Gy for *in vivo*)^44^,^45^, we did not detect the activation of Smad2 in keratinocytes irradiated with X-rays up to 10 Gy (Fig. 3d). The possible explanation for the discrepancy may be the cell type dependence of Smad2 activation. Interestingly, accompanied with lack of activation of Smad2 in HaCaT cells irradiated with X-rays, there was no bystander micronucleus induction in unirradiated WS1 cells after co-culture with X-irradiated HaCaT cells, though X-rays at all doses caused more serious damage in HaCaT cells than 56 cGy of α-particles did (Fig. 2). These data suggest that the activation of TGF-β1-Smad2 pathway of HaCaT cells upon radiation but not cell killing may be essential for the bystander effect in unirradiated WS1 cells. It is still unknown what bystander signaling molecules can be released from α-irradiated keratinocytes following the activation of TGF-β1-Smad2 pathway. Recent study shows that there is some crosstalk between TGF-β and NF-κB pathways, and inhibition of the TGF-β1 signaling in primary keratinocytes suppresses UVB induction of TNF-α, a well characterized NF-κB target gene^47^. TNF-α has been demonstrated to be an important RIBE mediator in some cell type^48^. Whether TNF-α plays any role in RIBE in our system needs further investigation.

Profiling studies have demonstrated that miRNA expression levels change in response to ionizing radiation^49^. Moreover, the miRNA changes are radiation type and dose-specific^38^. However, it was still unclear what roles the miRNA changes induced by radiation play in RIBEs. Previous study shows that miR-663 is downregulated in irradiated cells and it inhibits bystander effects by interacts with TGF-β1 directly^33^. In this study, we found that α-irradiation induced consistent decrease in miR-21 expression in HaCaT cells while X-rays induced a decrease at first then an increase at later times (Fig. 4a). Interestingly, downregulating the miR-21 level in HaCaT cells alone could induce RIBE-like MN formation in WS1 cells using co-culture system (Fig. 4e). And upregulating the miR-21 level in HaCaT cells prior to α-irradiation could abolish the RIBE in WS1 cells after co-culture (Fig. 4c). These results suggest that miR-21 of irradiated HaCaT cells played an important role in RIBE, and more specifically, its downregulation was necessary for the occurrence of bystander effect.

Furthermore, we found that miR-21 and the TGF-β1-Smad2 pathway of α-irradiated HaCaT cells were not independent and they regulated each other. The relationship between miR-21 and TGF-β1 signaling appears to be bidirectional. On one hand, both Smad7 and TGFBR2 are among the targets of miR-21^29^,^40^. miR-21 regulates the expression of Smad7 by inhibiting the translation of its mRNA. Smad7 and TGFBR1 competitively bind to Smad2 and Smad3. Thus upregulated Smad7 by decreased miR-21 prevents the activation of Smad2 and Smad3^29^. The regulation of miR-21 on TGFBR2 leads to a different story. When the miR-21 level is reduced in cells, the TGFBR2 protein level increases followed by formation of heterodimers with TGFBR1 and activation of Smad2/3^40^. This agrees with our observation on the phosphorylation of Smad2 in cells with downregulated miR-21 (Fig. 5c). Moreover, when the miR-21 overexpressed HaCaT cells were irradiated by α-particles, the activation of Smad2 was inhibited (Fig. 5b). On the other hand, TGF-β1-Smad signaling can act as a critical upstream regulator of miR-21^41^,^42^,^50^,^51^. More specifically, miR-21 expression is positively regulated by TGF-β1-Smad3 and negatively regulated by TGF-β1-Smad2 via miRNA biogenesis^50^. In the present study, we found that when the TGF-β1-smad2 pathway was inhibited, the decrease in miR-21 in α-irradiated HaCaT cells were abolished or reversed (Fig. 5a). This fits well with the previous study^50^.

**Figure 6:**
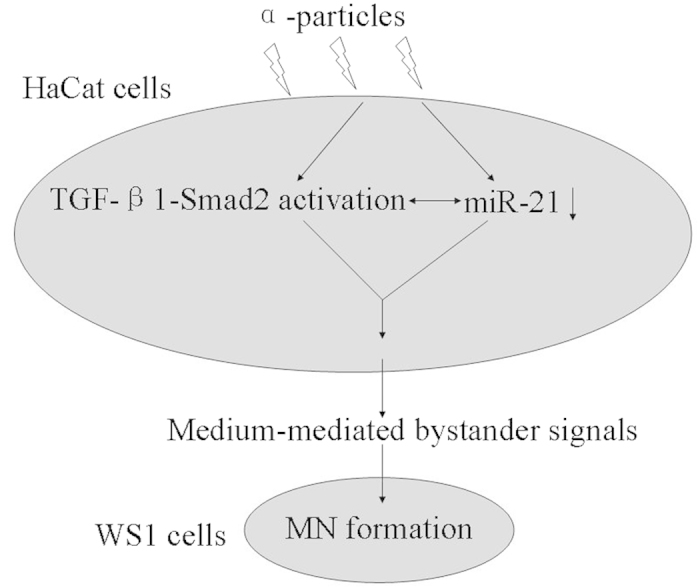
The working model of bystander MN formation in unirradiated WS1 cells after co-cultured with α-irradiated HaCaT cells.

In summary, using a transwell insert co-culture system to study the bystander MN formation in WS1 human skin fibroblasts induced by irradiated HaCaT keratinocytes, we found that the bystander effect was dependent on radiation quality. α-particles but not X-rays could induce bystander effect. More importantly, the activation of TGF-β1-Smad2 pathway and the consistent decrease in miR-21 level in α-irradiated HaCaT cells were essential to the increase in the MN frequency of bystander WS1 cells. And these two factors regulated each other to mediate the induction of bystander effect (Fig. 6). On the other hand, X-irradiation did not cause Smad2 activation and consistent decrease in miR-21 in HaCaT cells, neither did induce bystander effect in unirradiated WS1 cells. Taken together, these results suggest that the radiation quality-dependence of bystander effect may be associated with the TGF-β1-Smad2 pathway and the change of the miR-21 level in irradiated cells.

## Materials and Methods

### Cell culture and co-culture system

The human immortalized keratinocytes HaCaT cells were obtained from China Center for Type Culture Collection (CCTCC, Wuhan, China). The human embryonic dermal fibroblasts WS1 cells were obtained from American Type Culture Collection (ATCC, Manassas, VA, USA). Both cell lines were grown in DMEM (high glucose, Sigma-Aldrich, St Louis, MO, USA) supplemented with 10% fetal bovine serum (FBS, Wisent, St-Bruno, Quebec, Canada), 100 U/ml streptomycin and 100 U/ml penicillin (both from Beyotime Institute of Biotechnology, China) at 37 ^°^C in a humidified atmosphere of 95% air and 5% CO_2_.

To study the factors in irradiated HaCaT cells that were involved in the bystander effect in unirradiated WS1 cells, a transwell insert co-culture system was utilized. 1.2 × 10^5^ HaCaT cells and 4 × 10^4^ WS1 cells were seeded on coverslips in 6-well plates (with a growth area of 9.6 cm^2^) and in companion Millicell^®^ transwell culture inserts (Millipore, MA, USA) (with a growth area of 5.7 cm^2^), respectively. The insert had a porous membrane with a pore size of 0.4 μm to allow the passage of molecules but not cells. Immediately after HaCaT being irradiated, the inserts with WS1 cells were put into the wells with HaCaT cells for 24 h. HaCaT and WS1 cells shared the same medium, but were separated from each other with a distance of 3 mm.

### Irradiation

Twenty-four hours after plating, HaCaT cells on coverslip were irradiated with 56 cGy of alpha particles emitted from the ^241^Am source of an α-irradiation equipment at a dose rate of 0.14 Gy/min as described previously^52^. For X-irradiation, HaCaT cells were replenished with fresh medium immediately before irradiation, then they were irradiated with 160 kVp X-rays (RAD SOURCE RS2000 X-ray machine, USA) at a dose rate of 1.16 Gy/min. The LET values of α particles and X-rays are about 95 and 2 keV/μm, respectively.

### Micronuclei assay

For irradiated HaCaT cells, 48 h after irradiation, the cells were fixed with methanol: acetic acid (3 : 1, v/v). For bystander WS1 cells, 24 h after co-cultured with irradiated HaCaT cells, WS1 cells in inserts were fixed using the same method. After air drying, the cells were rehydrated and stained with 5 μg/ml of 4’, 6’-diamidimo-2-phenylindole (DAPI, Beyotime, China). The nuclei with micronucleus were counted under a fluorescent microscope (Leica DM 2000, Germany). At least 2000 cells were examined for each sample.

### Clonogenic assay

Immediately after irradiation, HaCaT cells were trypsinized and seeded in 60 mm petri dishes at different density depending on the radiation dose. The cells were then kept in incubator for 14 d before fixation with methanol and staining with methylene blue. The colonies with more than 50 cells were counted and the percentage of cell survival was calculated.

### microRNA extraction and Real time PCR

At various times, irradiated HaCaT cells were collected and the microRNA was isolated and purified using the E.Z.N.A.^TM^ miRNA Kit (Omega Bio-Tek Inc., USA). Reverse transcription and quantitative real-time PCR were subsequently performed using the TaqMan^®^ MicroRNA Reverse Transcription Kit and the TaqMan^®^ MicroRNA Assays (AB Applied Biosystems, USA) as described previously^53^. The PCR results were normalized with the internal control, RNU6B. And the expression of miR-21 in the untreated control was set as 1 to generate the relative expression level in the treated cells.

### Western blot analysis and antibodies

The expression of phosphorylated Smad2 and total Smad2 was detected by western blotting. In brief, HaCaT cells were lysed in lysis buffer containing 0.1% Triton X-100, 10 mM Tris (pH7.4), 10% glycerol, 150 mM NaCl, 5 mM EDTA, 1 mM sodium orthovanadate, 1 mM phenylmethylsulfonyl floride (PMSF), and 0.1% complete protease inhibitor cocktail. The proteins were separated on a 10% SDS-polyacrylamide gel and transferred to a polyvinylidene difluoride membrane (PVDF, BioRad, Hercules, CA, USA). The blots were probed with rabbit anti-phospho-Smad2 (Ser465/467) mAb (1:1000, Cell Signaling Technology, Inc., Beverly, MA, USA), rabbit anti-Smad2 mAb (1:10000, Abcam, UK) or mouse anti-β-actin mAb (1:1000, Beotime, China) followed by goat anti-rabbit IgG-horse radish peroxidase-conjugated (HRP) antibodies (1: 1000, Beyotime, China) or goat anti-mouse IgG HRP anitbodies (1: 1000, Beyotime, China). ECL kit (BioRad, USA) was used for chemiluminescent visualization of proteins on Typhoon 9410 high performance gel and blot imager (GE Amersham, USA). β-actin was used as loading control.

### TGF-β1 signaling inhibition with SB431542

SB431542 (Sigma-Aldrich, USA), a potent and selective inhibitor of TGF-β1 receptor kinases was used to inhibit the TGF-β1 pathway in irradiated HaCaT cells. SB431542 was added to HaCaT cells at a final concentration of 10 μM 1 h prior to irradiation, immediately before radiation, the medium was replaced with fresh medium. WS1 cells were then put into co-culture with irradiated HaCaT cells in the fresh medium without SB431542.

### Transfection of miR-21 mimic and inhibitor

The miR-21 mimic (5’-UAGCUUAUCAGACUGAUGUUGAAACAUCAGUC UGAUAAGCUAUU-3’) and its negative mimic (NC, sense 5’-UUCUUCGAACG UGUCACGUTT-3’ and antisense 5’-ACGUGACACGUUCGGAGAATT-3’), or the miR-21 inhibitor (IN, 5’-UCAACAUCAGUCUGAUAAGCUA-3’) and its negative inhibitor (IN.NC, 5’-CAGUACUUUUGUGUAGUACAA-3’) were purchased from GenePharma (Shanghai, China). When HaCaT cells got 70% confluent, they were transfected by adding the mixture of the miR-21 mimic or inhibitor (0.3 μM) and lipofectamine^®^ 2000 (Life Technologies, USA) according to the manufacturer’s instructions. Six hours after transfection, the transfection medium was replaced with fresh medium, and the cells were used at different times depending on the purpose.

### Statistical analysis

All data in this paper are presented as the average of at least three independent experiments  ±  standard error (SE). Differences between the control group and the treated group were analyzed using the Student’s t test of Origin 8 software. A P value of <0.05 between groups was considered significantly different.

## Additional Information

**How to cite this article**: Yin, X. *et al.* Radiation quality-dependence of bystander effect in unirradiated fibroblasts is associated with TGF-ß1-Smad2 pathway and miR-21 in irradiated keratinocytes. *Sci. Rep.* **5**, 11373; doi: 10.1038/srep11373 (2015).
